# Molecular mechanisms and energetic costs of recovery from freezing in the polyextremophile midge *Belgica antarctica*

**DOI:** 10.1242/jeb.252542

**Published:** 2026-08-12

**Authors:** Cleverson Lima, Melise C. Lecheta, Samuel Cecconi, Scott Hotaling, Paul B. Frandsen, Peter Convey, Scott A. L. Hayward, J. D. Gantz, Yuta Kawarasaki, Jeramiah J. Smith, Nicholas M. Teets

**Affiliations:** ^1^ Department of Entomology, University of Kentucky, Lexington, KY 40546-0091, USA; ^2^ Department of Biology, University of Kentucky, Lexington, KY 40508-0225, USA; ^3^ Department of Watershed Sciences, Utah State University, Logan, UT 84322, USA; ^4^ Department of Plant and Wildlife Sciences and Bean Life Science Museum, Brigham Young University, Provo, UT 84602, USA; ^5^ British Antarctic Survey, Natural Environment Research Council, Cambridge, CB3 0ET, UK; ^6^ Department of Zoology, University of Johannesburg, Auckland Park 2006, South Africa; ^7^ Millennium Institute - Biodiversity of Antarctic and Sub-Antarctic Ecosystems (BASE), 3425 Santiago, Chile; ^8^ School of Biosciences, University of Birmingham, Birmingham, B15 2TT, UK; ^9^ Department of Biology and Health Sciences, Hendrix College, Conway, AR 72032, USA; ^10^ Department of Biology, Gustavus Adolphus College, Saint Peter, MN 56082, USA

**Keywords:** Extreme adaptations, Energy reserves, Anti-apoptosis, Autophagy, Heat shock proteins, Cytoskeleton remodeling

## Abstract

Extracellular freezing challenges insects in several ways, including mechanical damage from ice crystals, exposure to stressful levels of cold and cellular dehydration. While stress-response mechanisms activated during and immediately after freezing are well studied, less is known about how freeze-tolerant organisms recover from freezing in the longer term and whether this recovery carries energetic costs. Here, we tracked changes in gene expression and energy stores over the course of 15 days following extracellular freezing using the world's southernmost insect, *Belgica antarctica* (Diptera: Chironomidae), as a study system. We found that *B. antarctica* employed a coordinated ‘emergency’ stress-response system during early recovery (0–1 day), which includes the upregulation of genes involved in inhibiting cell death (e.g. Bcl-2, IAP-1) alongside mechanisms involved in damage repair and clearance of cell debris (e.g. heat shock proteins, autophagy, proteasome). Concomitantly, genes involved in ecdysone biosynthesis and juvenile hormone degradation were downregulated through the 1st day of recovery, and several genes involved in cuticle development were downregulated between the 3rd and 15th day of recovery, suggesting that homeostasis may not have been completely restored for 2 weeks after *B. antarctica* had thawed. These physiological changes did not lead to a detectable depletion of energy stores, indicating that this emergency response system does not incur significant energy drain. These results suggest that evoking a coordinated stress response with minimal energy usage may be crucial for *B. antarctica* to persist in cold environments, and that blocking apoptosis and pausing development could provide sufficient time for freezing injury to be fully addressed.

## Teaser

**Summary:** The Antarctic insect *Belgica antarctica* recovers from freezing using minimal energy stores, potentially due to a sophisticated stress-response system that involves blocking cell death, repairing damage, suppressing metabolism and pausing development.

## INTRODUCTION

Insects that experience sub-zero temperatures are typically categorized into those that succumb to ice formation (i.e. freeze intolerant or freeze avoiding) and those that tolerate ice formation (i.e. freeze tolerant; Bale, 1993). For freeze-tolerant insects, the ability to repair injury and restore homeostasis is critical to enable resumption of development and other essential functions after thawing. The physicochemical effects of freezing, such as modifications of macromolecule composition, mechanical damage caused by ice formation and disruption of cell integrity caused by cellular dehydration are well established (Storey and Storey, 2013; Toxopeus and Sinclair, 2018; Rozsypal, 2022), and the mechanisms used by insects to prepare for freezing through acclimation are also well characterized (e.g. Des Marteaux et al., 2019; Toxopeus et al., 2019). However, the mechanisms by which insects repair injury and recover physiological functions post-thaw have received less attention. Therefore, investigating how insects recover from freezing, and its long-term costs, is critical for a more comprehensive understanding of insect freeze tolerance.

Extracellular ice formation causes numerous physiological disruptions that must be reversed during recovery (reviewed by Rozsypal, 2022; Toxopeus and Sinclair, 2018). Ice crystallization typically initiates in extracellular spaces, which increases solute concentration (i.e. ‘freeze concentration’) as water in the hemolymph freezes and causes intracellular water to diffuse out of cells, resulting in freezing-induced cellular dehydration (Lee, 1989). Ultimately, this osmotic stress requires redistribution of ions and water during recovery; for example, through the action of aquaporins, which are required for some insect cell types to survive freezing (e.g. fat body and midgut; Philip et al., 2008). Restoration of ion and water balance also involves maintaining or recovering the function of electrogenic ion pumps (such as the Na^+^/K^+^-ATPase; Koštál et al., 2004, 2006; MacMillan et al., 2015) and likely proteins involved in passive transport (such as Na^+^ and K^+^ leak channels; Beyenbach and Piermarini, 2008). Growth of ice crystals can cause mechanical damage to cells and tissues depending on their shape and location (Storey and Storey, 1988; Sinclair and Renault, 2010), which may necessitate repair or replacement of biological membranes and macromolecules associated with these membranes. Freezing can also cause the formation of cytotoxic aggregates of misfolded proteins (Korsloot et al., 2004) that either need to be refolded (Feder and Hofmann, 1999) or moved to the proteasome for degradation (Kubota, 2009). Because oxygen does not diffuse well through ice (Scholander et al., 1953), oxygen availability and transport are likely reduced during freezing, which may promote the accumulation of harmful metabolites as a consequence of a shift to anaerobic metabolism (Storey and Storey, 2013). Moreover, thawing leads to rapid reoxygenation in cells and tissues, which can also cause oxidative stress through the accumulation of harmful metabolites and build-up of reactive oxygen species (Doelling et al., 2014; Štětina et al., 2018; Storey and Storey, 2013). Repairing damage caused by extracellular freezing requires de novo production of proteins and other macromolecules, active transport and catabolism of waste products, all of which are energetically demanding (Lynch and Marinov, 2015; Nicholls, 2013). Thus, the energetic demands of recovering from a freezing event could ultimately lead to fitness consequences.

Challenges associated with freezing are particularly relevant in Antarctica, the coldest continent on the planet, where temperatures below zero can occur at any time of the year (Convey et al., 2018; Kawarasaki et al., 2014a). Terrestrial invertebrate diversity is extremely low relative to the rest of the planet (Chown and Convey, 2016; Convey and Biersma, 2023), with freezing and other abiotic challenges representing important barriers to species establishment. Only two species of free-living insects are native to the Maritime Antarctica: the winged midge, *Parochlus steinenii*, and the wingless midge, *Belgica antarctica*, both dipterans from the Chironomidae family (Convey and Block, 1996; Chown and Convey, 2016). Additionally, a few insect species have been introduced into Antarctica in recent years, including the chironomid midge *Eretmoptera murphyi* (Diptera: Chironomidae), the cranefly *Trichocera maculipennis* (Diptera: Trichoceridae) and the moth fly *Psychoda albipennis* (Diptera: Psychodidae), all of which are either established or in the process of becoming established in the South Orkney and South Shetland Islands (Bartlett et al., 2023; Maturana et al., 2024; Hughes et al., 2013, 2025). Thus, relatively few insect species can make Antarctica home, primarily due to the prolonged periods of subzero temperatures and short growing seasons (Chown and Convey, 2016).

*Belgica antarctica* is the world's southernmost distributed insect and is uniquely adapted to Antarctica's ephemeral environments. This species is found predominantly on offshore islands and in the mainland throughout the western Antarctic Peninsula, as well as in the South Shetland Islands (Usher and Edwards, 1984; Kovalenko et al., 2021). The larvae feed on dead plant material, algae and microorganisms during their typical 2 year life cycle (Nardi et al., 2009; Sugg et al., 1983). *Belgica antarctica* larvae have a patchy distribution but are abundant where they occur (ca. 40,000 individuals per square meter; Rico and Quesada, 2013; Potts et al., 2020), likely playing an important role in nutrient cycling, as has been documented in the sister species *E. murphyi* (Bartlett et al., 2023). The wingless adults emerge synchronously during the Austral summer (ca. December–March), reproduce and die within 14 days (Sugg et al., 1983; Usher and Edwards, 1984). Freezing significantly influences life cycle synchronization in *B. antarctica*, and 4th-instar larvae appear to undergo an obligate diapause that requires prolonged freezing for termination (Yoshida et al., 2025). In dry microclimates, these insects can avoid freezing (Elnitsky et al., 2008), but the typically moist microhabitats, coupled with a high susceptibility to inoculative freezing, mean that Antarctic insects are likely to be frozen at sub-zero temperatures on a regular basis in their natural habitats (Kawarasaki et al., 2014a). The ability to readily repair injury caused by internal ice formation and recover from freezing is, therefore, critical for *B. antarctica*’s success.

Although long-term effects of exposure to freezing remain unclear, short-term changes have been investigated extensively in *B. antarctica* larvae. For individuals collected in summer (referred to as summer larvae), freezing is energetically costly, with frozen larvae having lower lipid and carbohydrate stores than supercooled larvae (i.e. exposed to the same sub-zero temperatures but not frozen) following recovery (Teets et al., 2011). Paradoxically, this energy drain during recovery is accompanied by reduced oxygen consumption (Teets et al., 2019), suggesting that freezing elicits an increased usage of less efficient anaerobic processes. However, winter larvae (previously referred as ‘winter acclimated’, ‘winter acclimatized’ or ‘cold acclimated’) are more freeze tolerant (Lee et al., 2006), and have reduced short-term energetic costs, with freezing only resulting in a slight reduction in glycogen (Teets et al., 2020). Likewise, when larvae experience gradual cooling and prolonged freezing (∼30 days), there is a modest reduction in glycogen stores, but levels are re-established during recovery (Kawarasaki et al., 2014b). At the molecular level, recovery from freezing elicits increased expression of heat shock proteins (Teets et al., 2019, 2020), as well as differential expression of several genes involved in cryoprotectant synthesis and mobilization (Teets et al., 2013). However, energetic and molecular studies to date have only focused on immediate recovery (typically within 24 h), and there is a need to examine these processes over a longer period to fully capture the mechanisms involved in recovery from freezing and help us to infer potential fitness costs.

Here, we investigated long-term effects of exposure to acute freezing stress in *B. antarctica* by measuring changes in gene expression and energy stores during recovery from sub-lethal freezing. Maintaining this species through an entire generation to fully capture fitness costs is not currently possible (see Yoshida et al., 2025), so we monitored survival, gene expression and energy stores for a 15 day recovery period as a proxy for potential fitness consequences. We hypothesized that recovery from freezing is energetically costly, as consumption of energy stores would be required to support stress-response mechanisms that reverse the negative effects of extracellular freezing. Specifically, we predicted that energy stores would be spent in the earlier stages of recovery to re-establish homeostasis but would be replenished later in recovery, as larvae resume feeding and other essential functions. At the molecular level, we predicted upregulation of genes for repairing damaged macromolecules (e.g. chaperones to aid in protein refolding) and removing/clearing irreversibly damaged proteins (e.g. via ubiquitin-proteasome degradation) and organelles (e.g. via autophagy) early in recovery. Then, damaged macromolecules and cells would be replenished later in recovery. Moreover, as molecular stress responses often inhibit the cell cycle and developmental progression (Feder et al., 1992; Krebs and Feder, 1997), we predicted that recovery from freezing would disrupt development, which would be reflected by the shutdown of developmental genes early in recovery and subsequent resumption later in recovery.

## MATERIALS AND METHODS

### Insect collection and experimental conditions

*Belgica antarctica* Jacobs 1900 larvae were collected in February and March of 2020 on islands near Palmer Station (64°46′ S, 64°04′ W). Soil samples (i.e. substrate with food) containing *B. antarctica* at mixed developmental stages were transferred to a 4°C low-temperature growth chamber (LT-36VLC8, Percival Scientific, Perry, IA, USA) on the research vessel and transported by ship to California, then by ground transport to the University of Kentucky, all of which took approximately 6 weeks. Because activity in *B. antarctica* continues regardless of the time of day (Kobelkova et al., 2015), photoperiod was not controlled but temperature was maintained at a constant 4°C. At the University of Kentucky, larvae were maintained in natural substrate in a controlled environment room (Environmental Growth Chambers, Chagrin Falls, OH, USA) for approximately 12 weeks. *Prasiola crispa*, a native Antarctic foliose alga, was available as a food source within the substrate, which was kept moistened by spraying with water regularly until experiments began in August 2020. Because *B. antarctica* overwinters as 4th instar larvae, we presumed that our specimens were likely in diapause (Yoshida et al., 2025).

Before exposure to freezing, 4th instar larvae were sorted from substrate in ice water and divided into experimental groups. Frozen groups were exposed to a sub-lethal dose of inoculative freezing (following Baust and Lee, 1987; Teets et al., 2020) by placing larvae in a 1.5 ml microcentrifuge tube containing approximately 50 μl water and a small piece of ice, then placing the tubes at −5°C for 24 h in a programmable water bath (Arctic A10 refrigerated circulator, ThermoFisher Scientific, Waltham, MA, USA). Control groups were maintained in substrate at 4°C for 24 h. For recovery, larvae from both groups were placed in Petri dishes with moist filter paper and live *P. crispa* as a food source. Treated and untreated animals were simultaneously sampled for analysis immediately after frozen larvae had thawed (i.e. minutes after removal from the water bath; 0 days) and at 1, 3, 7 and 15 days of recovery. The Petri dishes were vented every 1–2 days to prevent anoxia.

### Survival assays

Survival during the recovery period was monitored by briefly placing larvae from control and frozen groups at each time point in ice water under a S6D Stereo Microscope (Leica Microsystems, Boston, MA, USA), and larvae that could move spontaneously or in response to gentle prodding with blunt forceps were considered alive. We assessed survival of 25 larvae per replicate (*n*=5 replicates), per treatment, per time point (total *n*=1000 larvae). Importantly, survival data were obtained from independent treatment groups at each time point; that is, groups that had survival assessed at each time point were used for downstream analysis and distinct larvae were assessed for survival at subsequent time points. Thus, groups of larvae that were alive at each time point were weighed, placed in microcentrifuge tubes and frozen at −80°C for RNA sequencing and macronutrient quantification assays (see below).

Statistical analysis was conducted in R v.4.5.2 (http://www.posit.co/). Differences in survival between frozen and control groups were tested using a generalized linear model [GLM; *glm(x, family=binomial)*] in the package stats (in base RStudio). In this model, survival (alive or dead) was the response variable, and recovery time, treatment (frozen and control) and their interaction were the predictors. Data visualization was carried out using the package ggplot2 v.4.0.2 (Wickham, 2016).

### Transcriptomics

#### Genome sequencing, assembly and annotation

Prior to transcriptomics analysis, we sequenced, assembled and annotated *B. antarctica*’s genome. Given the low amount of purified high molecular weight (HMW) DNA from a single individual, we used the PacBio Ultra-low library prep protocol with the SMRTbell Express Template Prep Kit 2.0 (PacBio, Menlo Park, CA, USA). In brief, HMW DNA was sheared to 10 kbp fragments using a Diagenode Megaruptor (Diagenode, Denville, NJ, USA) followed by whole-genome amplification and then a standard library preparation was carried out. Next, the ultra-low library was prepared and sequenced on a PacBio Revio flow at the Brigham Young University DNA Sequencing Center. To prepare the circular consensus sequencing (CCS) reads for assembly, we first trimmed PCR adapters and then removed PCR duplicates with lima (https://github.com/PacificBiosciences/barcoding/). We then assembled the trimmed and de-duplicated HiFi reads using hifiasm v.0.9.14 (Cheng et al., 2021). Genome completeness was evaluated with compleasm v.0.2.6 (Huang and Li, 2023) using the insecta_odb10 BUSCO (benchmarking universal single-copy orthologs) gene set.

The genome was annotated using the BRAKER3 pipeline (https://github.com/Gaius-Augustus/BRAKER; Brůna et al., 2025; Javor, 2025). BRAKER3 combines GeneMark-ETP (Brůna et al., 2020; Lomsadze et al., 2014) and AUGUSTUS (Stanke et al., 2008, 2006) to predict genes with high support from extrinsic evidence (i.e. RNA-seq and protein data). The RNA-seq data used for annotation (option --rnaseq_sets_ids) were obtained from the National Center for Biotechnology Information (NCBI) Genome Database (Table S1). Predicted protein sequences (option --prot_seq) were obtained from closely related chironomids and other well-studied dipterans published on NCBI (Table S2). Repeated regions were masked prior to annotation using RepeatMasker v.4.1.8 (http://www.repeatmasker.org). The outputs generated by BRAKER3 used in this study include the genomic features (.gff3; used for transcriptome assembly), the predicted protein sequences (.aa.fa; used for functional annotation) and predicted coding sequences (.codingseq; used to extract the length of each gene). This pipeline was conducted on the Morgan Compute Cluster (University of Kentucky). Functional annotation of BRAKER3 predicted proteins was conducted using eggNOG-mapper v.5.0.2 (Huerta-Cepas et al., 2017) implemented in Galaxy Web Service (https://usegalaxy.org/). Most default options were used, with the exception of --tax_group, which we set for the Arthropoda taxonomic group (taxID: 6656) and enabling the option --report_orthologs, which provided the orthologs used for annotation. This method generated functional description inferred for each gene and their orthologs, as well as gene to gene ontology (GO) and gene to Kyoto encyclopedia of genes and genomes (KEGG) orthology (KO) mapping for downstream analysis. The complete output from eggNOG-mapper can be found in Dataset 1.

#### RNA sequencing

Following survival assessments, larvae that were alive at each time point were placed in sample vials (10 larvae per replicate; *n*=4 replicates per treatment and time point; total *n*=400 larvae), snap frozen in liquid nitrogen and stored at −80°C until extraction. RNA was extracted using a modified protocol of the RiboPure RNA Purification Kit (ThermoFisher Scientific). Briefly, larvae were homogenized in TRI Reagent^®^ RNA Isolation Reagent (Sigma-Aldrich, Burlington, MA, USA), incubated at room temperature for 5 min and centrifuged at 4°C. The supernatant was transferred to clean tubes, then 1-bromo-3-chloropropane was added for phase separation, and the upper aqueous phase was removed. Isopropanol was added to precipitate RNA, and the RNA pellet was washed with 70% ethanol. The pellets were dissolved in nuclease-free water, and RLT buffer (Qiagen, Germantown, MD, USA) was added to each sample along with 100% ethanol. The RNA was then further purified using the RiboPure kit, according to the manufacturer's protocol. Samples were submitted for RNA sequencing to Novogene (San Diego, CA, USA), where preliminary purity and quantity of the samples were checked using NanoDrop, agarose gel electrophoresis and an Agilent 2100 bioanalyzer. Then, poly(A) mRNA enrichment and library preparation were conducted. Library quality control (QC) was conducted with a 2100 Bioanalyzer and qPCR, which checked for read insert sizes and library concentration, respectively. Libraries that passed QC were paired-end sequenced (2×150 bp) on a NovaSeq 6000 sequencer.

The obtained reads were further cleaned by removing adapters, removing reads containing undetermined bases (*N*>10%) and removing low-quality reads (Qscore≤5) in fastp (Chen et al., 2018), and final quality control was conducted in FastQC v.0.11.9 (https://www.bioinformatics.babraham.ac.uk/projects/fastqc//). Reads that passed quality control were aligned to *B. antarctica*’s reference genome (see above) using HISAT2 v.2.2.1 with default parameters (Kim et al., 2019). The resulting sequence alignment map files were sorted using Samtools v.1.13 (Danecek et al., 2021). Then, sorted samples were processed in StringTie v.2.1.7 (Pertea et al., 2016) using the genomic features file generated by BRAKER3 (see above) as a guide. A gene count matrix for differential expression analysis was generated using the prepDE.py script provided in the StringTie's manual (https://ccb.jhu.edu/software/stringtie/index.shtml?t=manual). This pipeline was carried out on the Morgan Compute Cluster (University of Kentucky).

#### Differential expression and enrichment analysis

The differential expression and enrichment analysis was conducted in R v.4.5.2 (http://www.posit.co/). Differential gene expression between frozen and control groups at each time point was conducted using the DESeq2 package v.1.50.2 (Love et al., 2014). Genes with *q*<0.05 (i.e. false discovery rate-adjusted *P*<0.05) were considered differentially expressed genes (DEGs) (Stephens, 2017). Following log-fold change shrinkage, which was carried out using the *lfcShrink()* function implemented in the ashr package v.2.2 (Stephens, 2017), we generated volcano plots for data visualization using the EnhancedVolcano package v.1.28.2 (https://github.com/kevinblighe/EnhancedVolcano). To identify biological processes potentially associated with recovery from freezing, we conducted GO (Ashburner et al., 2000; Aleksander et al., 2023) and KEGG (Kanehisa et al., 2017) analysis. GO overrepresentation analysis was conducted using the package goseq v.1.62.0 (Young et al., 2010), and KEGG analysis was conducted using the package gage v.2.60.0 (Luo et al., 2009). Visuals were generated using the packages ggplot2 v.4.0.2 (Wickham, 2016) and pathview v.1.50.0 (Luo and Brouwer, 2013).

### Macronutrient assays

Macronutrients from larvae that were alive at each time point were quantified following Spacht et al. (2021). For each assay, we used 5 replicate pools (5 larvae per replicate), per treatment, per time point (total *n*=50 pooled samples per macronutrient) and protocols are summarized below.

To measure carbohydrate content, larvae were homogenized in phosphate-buffered saline containing 0.05% Tween and centrifuged (14,000 ***g*** at 4°C for 5 min). The supernatant was transferred to a clean vial and centrifuged a second time (14,000 ***g*** at 4°C for 5 min) to clarify the samples. Anthrone reagent (1.4 mg anthrone ml^−1^ in 68% sulfuric acid) was added to all samples and standards, and they were vortexed and heated for 17 min at 100°C. Aliquots of each sample and standard were then transferred in triplicate to 96-well plates, and absorbance at 625 nm was measured on a spectrophotometer (Clariostar multimode plate reader, BMG Labtech, Cary, NC, USA). Absorbance of samples was compared with a 7-point standard curve prepared using 0, 250, 500, 750, 1000, 1500 and 2000 μg ml^−1^ glucose.

To measure lipid content, larvae were homogenized in 2:1 chloroform:methanol and centrifuged (13,000 ***g*** at 4°C for 5 min), and the supernatant was transferred to a clean glass test tube. Following the preparation of standards, which consisted of canola oil dissolved in chloroform, tubes were heated at 100°C to evaporate the solvent. We then added sulfuric acid and vortexed, followed by heating for 10 min at 90°C. Vanillin reagent (1.2 mg vanillin ml^−1^ in 68% phosphoric acid) was added to each sample, and samples were kept in the dark at room temperature for 20 min to develop color before being transferred to a 96-well plate and measured in the spectrophotometer. The absorbance of the plate was read at 525 nm and compared with a 7-point standard curve prepared using 0, 25, 50, 100, 150, 200 and 300 μg of canola oil.

To measure total protein content, larvae were homogenized in radioimmuniprecitation assay (RIPA) buffer (ThermoFisher Scientific) and centrifuged (14,000 ***g*** at 4°C for 5 min). The samples were vortexed to resuspend the pellet, agitated on ice, centrifuged again (14,000 ***g*** at 4°C for 5 min), and the supernatant transferred to new vials. The vials were centrifuged again (14,000 ***g*** at 4°C for 5 min) to clarify the solutions. The samples and standards were pipetted (in triplicate) into a 96-well plate, and bicinchoninic acid reagent (Pierce BCA Assay Kit, ThermoFisher Scientific) was added to each well. The well plate was briefly agitated, then incubated in the dark for 30 min at 37°C to develop color before being transferred to the spectrophotometer. The absorbance was read at 563 nm and compared with a 7-point standard curve prepared using 0, 125, 250, 500, 1000, 1500 and 2000 μg ml^−1^ albumin.

Statistical analysis was conducted in R v.4.5.2 (http://www.posit.co/). Changes in energy stores throughout the recovery period between frozen and control groups were analyzed using linear models [LMs; *lm()*] in base R. In our models, macronutrient content was included as the response variable, whereas recovery time, treatment and their interactions were used as predictors. The estimated dry mass was included in our models as a covariate to account for differences in mass across replicates. We estimated the dry mass by multiplying the fresh mass by 0.27, which was based on the average body water content (i.e. 73% of total mass) of fully hydrated *B. antarctica* larvae (e.g. Baust and Edwards, 1979; Benoit et al., 2009; Teets et al., 2012). Model diagnostics were conducted using the function *plot()* in base R. Because our working hypothesis assumed non-linearity (i.e. macronutrient content could change in either direction at any time point), recovery time was treated as a categorical predictor. To test the overall effect of time on macronutrient content between frozen and control, a Type 3 ANOVA was carried out on each model using the *Anova()* function from the car package v.3.1 (Fox and Weisberg, 2018) and Tukey HSD (emmeans v.2.0.2; https://CRAN.R-project.org/package=emmeans) was conducted as a *post hoc* test to further examine differences in macronutrient content between time points. Data visualization was generated using the ggplot2 package v.4.0.2 (Wickham, 2016).

The original experiment was designed assuming that *B. antarctica* would have the opportunity to feed when active, and thus we provided food throughout the experiment. In the following year, we carried out a second experiment to investigate whether the inability to feed would influence the observed results (see Results). In this experiment, we repeated the protocol described above, with the exception that larvae were only sampled at 1, 7 and 15 days following exposure to freezing.

## RESULTS

### Survival

**Fig. 1.**
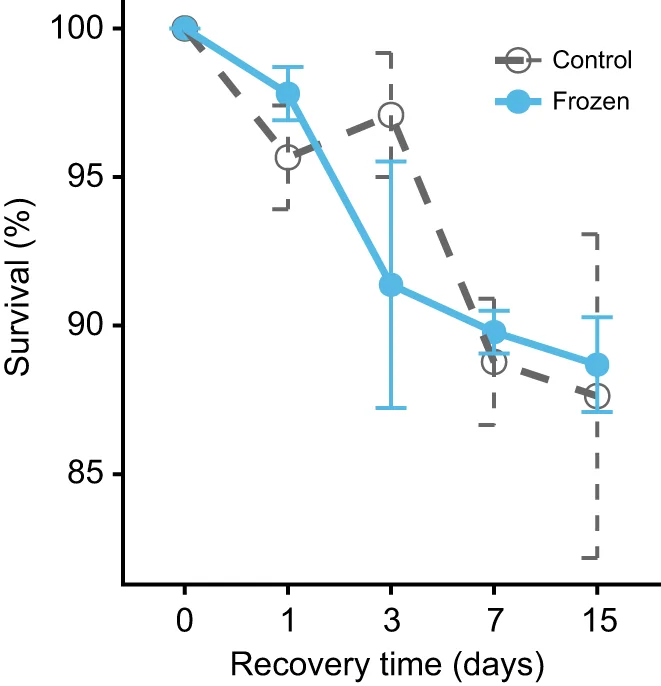
**Survival of *Belgica antarctica* larvae during recovery from freezing.** Larvae were either frozen at −5°C or maintained at 4°C for 24 h and survival was assessed at 0, 1, 3, 7 and 15 days following treatment. We assessed survival of 25 larvae per replicate (*n*=5 replicates), per treatment, per time point (total *n*=1000 larvae). The plot shows the mean±s.e.m. proportion (displayed as a percentage) of larvae that survived to treatment at each time point. A significant difference in survival was observed over time (GLM, *P*<0.05), but not between frozen and control, and there was no significant interaction between time and treatment (GLM, *P*>0.05).

The proportion of larvae that survived varied between 100% and 87% throughout the recovery period, with the lowest mean (±s.e.m.) survival recorded on day 15 for both treatments (i.e. 88±5% for frozen larvae and 87±1% for untreated controls). A significant difference in survival was observed over time (GLM, *P*<0.001; Fig. 1), but there was no difference detected between frozen and control or a significant interaction between recovery time and treatment group (GLM, *P*>0.05; Fig. 1). In a second experiment, in which larvae recovered in the absence of food, there was similarly no difference in survival between frozen and control larvae (Fig. S1).

### Genome assembly and annotation

The genome assembly was evaluated with BUSCO (v.5.8.3) in --genome mode against the Insecta lineage dataset (*n*=1332). The analysis identified 99% complete BUSCOs, with 97.4% found as single-copy and 1.2% as duplicated. Only 0.29% were fragmented, and 1.02% were missing, indicating a high-quality and near-complete genome suitable for RNA sequencing and functional annotation (Table S3). Additionally, the contig N50 (3.2 Mbp) represented multiple orders of magnitude improvement in contiguity from the previous *B. antarctica* genome assembly (N50: 13.7 kbp, NCBI accession: GCA_000775305.1).

Approximately 85.13% of the predicted protein sequences from our genome (*N*=13,999/16,444) matched the NCBI arthropoda database through eggNOG. The annotation statistics are summarized in Table S1, and the full annotation used in downstream analysis is presented in Dataset 1.

### Changes in gene expression induced by extracellular freezing

RNA sequencing generated approximately 51 million reads per sample. Approximately 4% of the total number of reads did not pass quality control and were removed, resulting in ca. 1 billion paired-end 150 bp reads. The reads were mapped to *B. antarctica*’s genome with an approximate 95% alignment rate (Table S3).

**Fig. 2.**
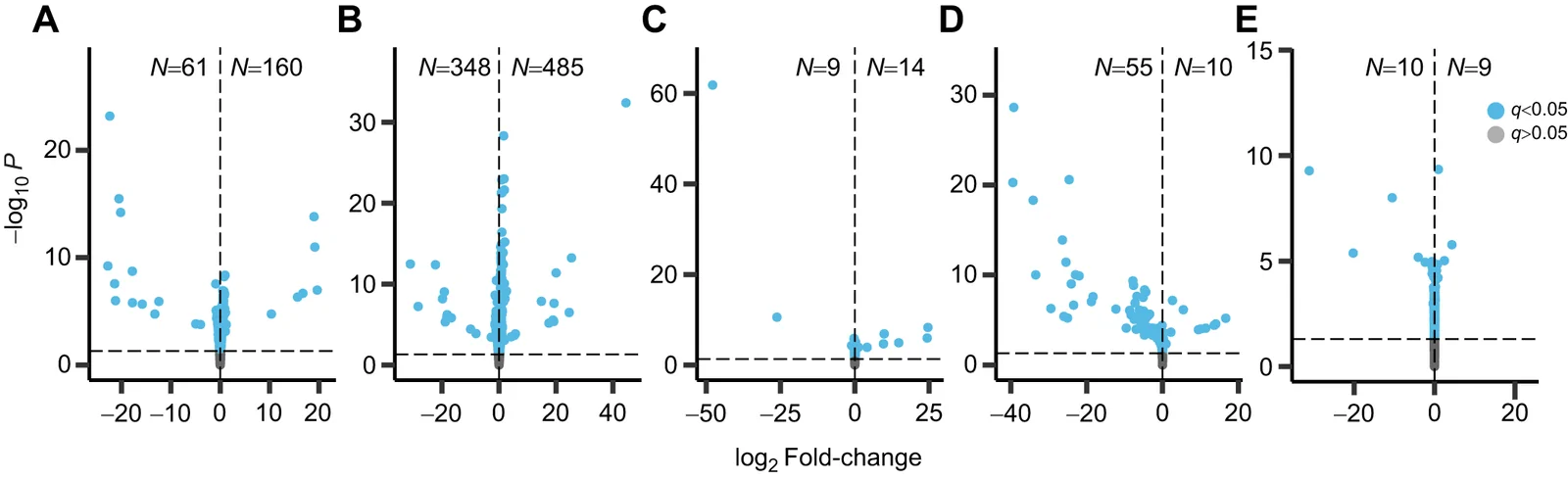
**Volcano plots indicating differentially expressed genes (DEGs) between frozen and untreated *B. antarctica* larvae throughout recovery from freezing.** Each plot represents one sampling time point (from A to E, 0, 1, 3, 7, 15 days post-exposure to −5°C for 24 h). RNA sequencing was carried out using 10 larvae per replicate; *n*=4 replicates per treatment, per time point; total *n*=400 larvae. Fold-change for each gene is displayed on a log_2_ scale (*x*-axis). Statistical significance of differential expression is shown as −log_10_*P* (*y*-axis). The horizontal dashed line represents the significance threshold. Blue dots represent genes that were differentially expressed between frozen and control, while gray dots represent genes with similar expression patterns between treatments. The numbers (*N*) within each plot represent the total number of downregulated and upregulated genes (*q*<0.05) at each time point.

Most DEGs in frozen relative to untreated larvae were upregulated or downregulated early in recovery (at time points 0 and 1 day), whereas fewer DEGs were observed later in recovery (i.e. between days 3 and 15; Fig. 2; Table S4). Some DEGs that were upregulated early in recovery included transcripts that code for heat shock proteins (e.g. *HSP20*, *HSP70* and *HSP90*), autophagy-related genes (e.g. *atg2*, *atg13*, *WIPI*, *SESN1*) and genes involved in cell death inhibition (e.g. *IAP1* and *Bcl-2*), whereas no pro-apoptotic genes were upregulated. Meanwhile, *JHEH2*, which codes for a juvenile hormone hydrolase, and *CYP302A1*, which codes for an intermediate in ecdysone biosynthesis, were downregulated early in recovery. Moreover, specifically at time point 1 day, several genes seemingly involved in carbohydrate metabolism (e.g. *UGP*, *PfkB*), lipid metabolism (e.g. *yip2*, *MOGT2*), amino acid metabolism (e.g. *Dip-C*) and ATP biosynthesis (e.g. *GPDH*) were downregulated. At the 3rd day of recovery, we observed the upregulation of *Hel25E*, *NHP2* and other genes involved in RNA cleavage and ribosome biogenesis, whereas all genes related to damage repair expressed in early-recovery returned to levels similar to those of untreated controls. At the 7th day of recovery, nearly 50% of the total downregulated genes (i.e. 25) were involved in cuticle structure and development, and three of these remained downregulated at day 15. The complete list of genes that were differentially expressed in each time point is given in Dataset 2.

Our KEGG analysis identified a few pathways potentially involved in stress response that were upregulated early in recovery (0–1 day), including Autophagy (ko04140), Fc gamma *R*-mediated phagocytosis (ko04666), Endocytosis (ko04144), Ubiquitin-mediated proteolysis (ko04120) and regulation of cytoskeleton (ko04810) (Figs S2 and S3). However, the proteasome pathway (ko03050) was downregulated at time point 0 days. Specific genes downregulated included those encoding structural and regulatory subunits (e.g. PSMA 1–7, PSMB 1–7, PSMC 1–6, PSMD 1–9, 12–14 and PSME 1, 3, 4; Fig. S4). At 1 day, several metabolic pathways were downregulated, including pathways involved in central carbon and energy metabolism (17), amino acid metabolism and biosynthesis (10), and lipid and fatty acid metabolism (6) (Fig. S2). Furthermore, 10 KEGG pathways associated with an increase in transcription and translation activity were upregulated at the 3rd day of recovery (Fig. S5). Examples include DNA replication (ko03030), RNA polymerase (ko03020), spliceosome (ko03013), nucleocytoplasmic transport (ko03013), as well as ribosome biogenesis (ko03008) and cell cycle (ko04110). No KEGG pathways were enriched at the 7th or 15th day (Table S4). The complete list of pathways that were enriched between frozen and untreated larvae for each time point is given in Dataset 2.

Our GO analysis generally agreed with results from KEGG analysis. For example, the GO terms Autophagy (GO:0006914), Autophagy of mitochondrion (GO:0000422), Endocytosis (GO:0006897), Ubiquitin-mediated proteolysis (GO:0030162) and Regulation of cytoskeleton organization (GO:0051493) were all overrepresented among upregulated genes. In addition, a number of GO terms involved in metabolism were overrepresented among downregulated genes at time point 1 day, including central carbon and energy metabolism (*N*=11), and lipid and fatty acid metabolism (*N*=19). On the 3rd day of recovery, rRNA pseudouridine synthesis (GO:0031118) and box H/ACA snoRNP complex (GO:0031429), both of which are involved in nucleic acid processing and ribosome biosynthesis, were overrepresented among upregulated genes. On day 7 of recovery, cuticle development (GO:0042335) and chitin-based cuticle development (GO:0040003) were overrepresented among downregulated genes. No GO terms were overrepresented at 15 days. The complete list of overrepresented terms at each time point is given in Dataset 2.

### Changes in energy stores following extracellular freezing

**Fig. 3.**
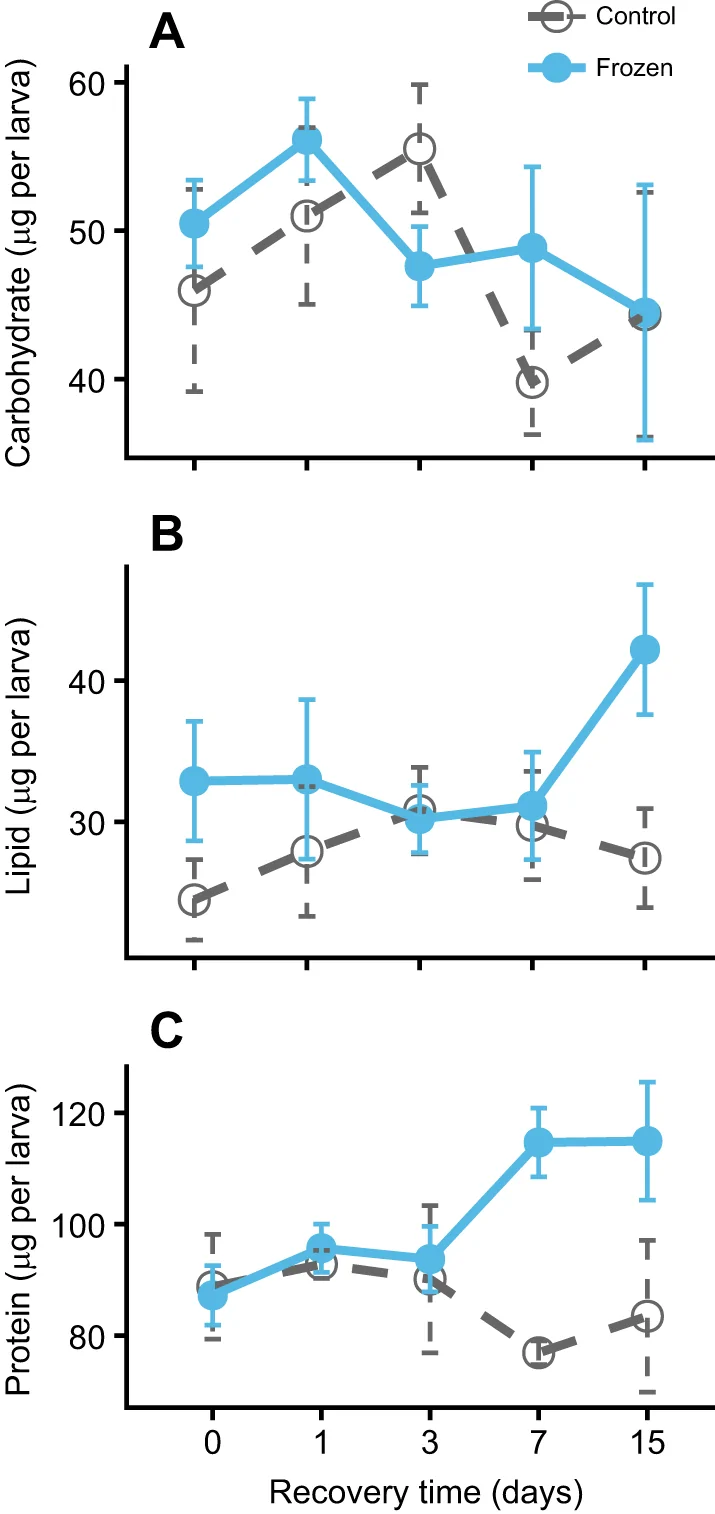
**Changes in energy stores during recovery of *B. antarctica* larvae from extracellular freezing.** Larvae were either frozen at −5°C or maintained at 4°C for 24 h and total macronutrient content was assessed at 0, 1, 3, 7, 15 days following treatment. For each assay, we used 5 replicate pools (5 larvae per replicate), per treatment, per time point (total *n*=50 pooled samples per macronutrient) and protocols. Plots indicate mean±s.e.m. (A) carbohydrate, (B) lipid and (C) protein. In our models, mass drove most of the effect on macronutrient content (GLM, ANOVA *P*<0.001), whereas no significant changes in macronutrient content were detected over time or between frozen and control (GLM, ANOVA *P*>0.05).

Macronutrient stores were generally similar between frozen and control larvae (Fig. 3). For example, mean (±s.e.m.) carbohydrate content per larva was 49.5±2.2 μg in frozen larvae and 47.3±2.7 μg in untreated larvae, which were not statistically different (LM, ANOVA, *P*>0.05; Fig. 3A). We also detected no trend of significant changes in carbohydrate or other macronutrient stores over time, nor a significant interaction between treatment and time (LM, ANOVA, *P*>0.05; Fig. 3). For all macronutrients, estimated dry mass of the sample was a significant predictor of content (LM, ANOVA, *P*<0.001). To determine whether macronutrient content may be influenced by changing body size across treatments, we analyzed the size of larvae for each set of samples (Fig. S6). In general, there was no difference in body size between frozen and untreated larvae, with the exception of samples from which protein content was measured, where the effect of treatment was significant at time points 7 and 15 days (Tukey HSD, *P*<0.05; Fig. S6C). However, we suspect this result was due to random sampling and not indicative of a true treatment effect as we found no differences in mass of larvae in which carbohydrates and lipids were measured (LM, ANOVA, *P*>0.05; Fig. S6C).

In the following year, we carried out a follow-up experiment to investigate whether the inability to feed would influence changes in macronutrient content during recovery from freezing. Similar to our findings above, macronutrient stores did not significantly change in frozen relative to untreated larvae (LM, ANOVA, *P*>0.05; Fig. S7). Carbohydrate and protein content were also similar over time (LM, ANOVA, *P*>0.05; Fig. S7A,C), but we detected a significant decrease in lipid content throughout the recovery period (GLM, ANOVA, *P*=0.041). Specifically, mean (±s.e.m.) lipid per larva was 14.1±1.0 μg at day 1, and decreased to 11.2±1.2 μg at day 15 of recovery (Fig. S7B). Lastly, no significant interaction between treatment and time was detected when food was not provided (LM, ANOVA, *P*>0.05; Fig. S7). For lipids and proteins, estimated dry mass was a significant predictor of content (LM, ANOVA, *P*<0.05). Mass did not significantly differ over time or between treatments in unfed larvae in which carbohydrates, lipids or proteins were measured (LM, ANOVA, *P*>0.05; Fig. S8).

## DISCUSSION

In this study, we tested the hypothesis that recovery from freezing would lead to long-term changes in gene expression and energy investment to reverse the cascade of physiological challenges that accompany extracellular freezing. To test this hypothesis, we assessed gene expression and energy stores over a 15 day period post-exposure to freezing. We observed a variety of subcellular processes activated during this period, but contrary to our prediction, there was no evidence of energy depletion at any point of recovery. Gene expression results indicated damage repair and metabolic suppression during the first day of recovery, consistent with prioritizing inducible stress responses at the expense of metabolism and development while freezing injury is being repaired. While gene expression largely returned to baseline conditions by the 3rd day, some genes involved in transcription and translation remained upregulated later in recovery while others involved in development (e.g. cuticular proteins) remained downregulated up to 15 days following thawing. In general, our results suggest that a short, non-lethal freezing event may cause considerable cellular injury, as it triggers the activation of a variety of repair mechanisms, while pausing development for several days, which could incur long-term consequences to fitness in this species. One limitation of our study is that we cannot differentiate between molecular responses to ice formation versus those involved in recovery from low temperature more broadly because all larvae were frozen during cold treatment. Nonetheless, larvae of *B. antarctica* are highly susceptible to inoculative freezing in their moist microhabitats (Kawarasaki et al., 2014a), so these experiments likely reflect recovery in typical field conditions. Below, we discuss our original predictions (see Introduction), other potentially novel mechanisms through which *B. antarctica* recovers from extracellular freezing, and the implications of these physiological processes for insect freeze tolerance.

### Sub-lethal freezing activates a generalized stress response

#### Repair of damaged proteins during early recovery

Our results indicate that exposure to freezing led to a heat shock response early in recovery in *B. antarctica*. Heat shock proteins aid in refolding damaged proteins and/or mediate their transport to the proteasome for degradation (Gething, 1997; Feder and Hofmann, 1999; Haslbeck and Vierling, 2015), and these results are consistent with previous work indicating a heat shock response during recovery from cold and freezing stress in multiple invertebrates (e.g. Agwunobi et al., 2021; Zhang et al., 2011), including *B. antarctica* (Teets et al., 2011, 2019, 2020). Despite the expected interaction between heat shock proteins and the proteasome during stress (Feder and Hofmann, 1999), at time point 0 days, KEGG analysis indicated downregulation of the proteasome pathway. Specific downregulated genes included structural and regulatory subunits, perhaps to prevent unnecessary protein degradation while proteins were being refolded. Another possible reason for downregulating the proteasome would be if protein synthesis was downregulated, thereby requiring less protein turnover, but we did not observe clear evidence of a shutdown of translation machinery. A potential explanation for this paradoxical upregulation of molecular chaperones coupled with decreased protein turnover is that refolding damaged proteins is more beneficial for energy conservation rather than investing in *de novo* protein translation. However, whether this is true warrants further investigation.

#### Removal and replenishment of irreversibly damaged proteins, organelles and cells

Processes involved in clearing cellular debris, such as autophagy, were upregulated early in recovery. Autophagy is a widely conserved degradation mechanism that disposes of damaged or unwanted cellular components, including organelles, via self-digestion (Glick et al., 2010; Tanida, 2011), which may be important when organelles become compromised by freezing stress (e.g. Collins et al., 1997). In *B. antarctica*, genes involved in autophagy are also upregulated in response to dehydration stress (Teets and Denlinger, 2013), and in the freeze-tolerant fly *Chymomyza costata*, there is evidence for upregulation of at least one autophagy-related gene during recovery from freezing (Štětina et al., 2018). Together, these studies suggest that increased expression of genes involved in autophagy could play an important protective role in freeze-tolerant insects, though whether this mechanism serves to clear specific components (e.g. mitochondria) or whether it is non-selective (e.g. digests any cellular component) is unclear. Further, autophagy may provide an additional benefit by serving as an emergency source of nutrients in a period of stress (e.g. Wu et al., 2011). After 1 day of recovery from freezing, autophagy activity was less clear, as one autophagy-related gene was downregulated (*atg13*) and one was upregulated (*DRAM*). However, other physiological processes involved in clearing cellular components were activated at this time point, including processes for removing macromolecules (e.g. Ubiquitin-mediated proteolysis and Endocytosis), strange bodies (e.g. Fc gamma R-mediated phagocytosis) and entire cells (e.g. apoptotic cell clearance).

Transmembrane proteins are susceptible to denaturation during prolonged exposure to low temperature (e.g. ion channels; Koštál et al., 2004, 2006; MacMillan et al., 2015), and so endocytosis could play a role in removing damaged proteins from biological membranes, which could then be transported to the proteasome for ubiquitin-mediated degradation (Kwon and Ciechanover, 2017). In *B. antarctica*, the ubiquitin-mediated proteasome is also upregulated in response to dehydration stress (Teets et al., 2012), and in other freeze-tolerant insects, protein degradation via the proteasome has been suggested as part of the molecular response to freezing injury (Des Marteaux et al., 2019; Štětina et al., 2018). Thus, it appears that clearing damaged macromolecules (e.g. transmembrane and cytosolic proteins) is critical during recovery from freezing, as it occurs across freeze-tolerant species. Notably, while the Proteasome KEGG pathway was upregulated on the 1st day of recovery, it was downregulated immediately following thawing (see above), perhaps due to a negative interaction between proteasome and autophagy signaling systems (Lőw et al., 2013).

#### Cytoskeletal remodeling during recovery from freezing

A variety of genes, GO terms and one KEGG pathway involved in cytoskeletal remodeling were all upregulated on the 1st day of recovery. Cellular dehydration caused by extracellular freezing likely disrupts cytoskeletal integrity by contracting cells (Toxopeus and Sinclair, 2018), and thus efficient damage repair and cytoskeletal turnover is potentially necessary to recover and maintain cellular structure. In freeze-tolerant *Gryllus veletis*, acclimation that confers freeze tolerance remodels the cytoskeleton (Toxopeus et al., 2019), which appears to improve tissue-specific protection against internal ice formation (van Oirschot and Toxopeus, 2025). Similarly, remodeling the cytoskeleton appears to be important for the malt fly *C. costata* to become freeze tolerant (Des Marteaux et al., 2018). In *B. antarctica*, cytoskeleton proteins are more abundant both during dehydration and rehydration relative to untreated controls (Li et al., 2009), and a similar pattern was found in response to freezing stress in this study. For example, the Regulation of cytoskeleton KEGG pathway was enriched, which included upregulation of cytoskeleton-specific chaperones and structural proteins. *Belgica antarctica* is freeze tolerant year around and does not require acclimation to survive freezing, so here, cytoskeletal remodeling appears to be a direct response to damage caused by freezing stress. Freeze tolerance in *B. antarctica* can be improved by acclimation and hardening (Lee et al., 2006; Teets et al., 2019), and it is possible that this plasticity in freeze tolerance would also involve preparatory cytoskeletal remodeling. Regardless, our results are consistent with a growing body of evidence that cytoskeletal remodeling is a critical component of both cold and freeze tolerance and across several taxa.

#### Anti-apoptotic signaling immediately upon thawing

Immediately upon thawing, *B. antarctica* appeared to inhibit programmed cell death via two distinct mechanisms, which may prevent unwanted cell death while injuries are repaired. The gene *IAP-1* (also referred as *Diap1* in insects) was upregulated at time point 0 days, which can suppress apoptosis by regulating the activity of several caspases (Deveraux and Reed, 1999; Doumanis et al., 2001; Wu et al., 2022). Another mechanism through which apoptosis is potentially blocked is via the *PI3K-Akt-mTOR* pathway, which signals cell growth and suppresses apoptosis in multiple organisms (e.g. Kennedy et al., 1997; Morris et al., 1996; Staveley et al., 1998). Here, we observed upregulation of the catalytic subunit of *PI3K* (*PI3KCA/B/D*), as well as associated genes involved in cell death inhibition, *Akt/PKB*, *SESN1*, *CREB3* and *Bcl-2/BNIP3*. *PI3K* stimulates synthesis of phosphatidylinositol (3,4,5)-trisphosphate, which, in turn, recruits *Akt/PKB*, resulting in cell growth and suppression of apoptosis (e.g. Kennedy et al., 1997). Although a direct link has not been made in insects, this signal could occur via *mTOR* activation (e.g. Lee et al., 2010) or increased activity of calcium regulators (e.g. *CREB3* and *Bcl-2/BNIP3*; Berridge et al., 1998; Burton et al., 2009; Field et al., 2018; Mirabelli et al., 2016). This shutdown of apoptosis during recovery is likely critical for providing sufficient time to repair freezing injury before damaged cellular components cause a positive feedback cascade of programmed and unprogrammed cell death.

There are a few routes through which freezing could promote apoptosis. For example, increased mitochondrial membrane permeability can release caspase-3 into the cytosol, which ultimately causes DNA fragmentation and initiates apoptotic signaling (Nössing and Ryan, 2022). In insects, freezing has been linked to mitochondrial damage and DNA fragmentation, as it causes mitochondrial swelling, membrane permeability changes and DNA fragmentation in non-freeze-tolerant phenotypes of the malt fly *C. costata*. However, this type of injury is not observed in freeze-tolerant flies (Štětina et al., 2020; Štětina and Koštál, 2024). Although it is uncertain whether there is a direct link between mitochondrial damage and DNA fragmentation in the context of freezing, it is possible that this type of injury is prevented by hiherto unknown physiological changes elicited during acclimation that confer freeze tolerance to *C. costata.* In *G. veletis*, acclimation that improves freeze tolerance involves upregulation of the apoptosis inhibitor IAP-1 (Toxopeus et al., 2019), providing further evidence that modulating apoptosis is an important aspect of surviving freezing. In chill-susceptible insects, shutdown of apoptosis has a well-established role in cold tolerance, as hardening that improves tolerance to cold shock is accompanied by a concomitant reduction in apoptotic activity (Yi et al., 2007; Yi and Lee, 2011). Together, it appears that negative regulation of apoptosis is an important aspect of cold survival across insects, and in *B. antarctica*, it appears that *PI3K-Akt-mTOR* signaling and/or upregulation of IAP-1 during early recovery plays a critical role.

### The effects of sub-lethal freezing on development

Our gene expression data suggest that freezing temporarily disrupts *B. antarctica*’s development via hormonal regulation. The gene *CYP302A1*, encoding an intermediate in ecdysone biosynthesis (Warren et al., 2002), was downregulated at the point of thawing (0 days), and *JHEH2*, encoding a hydrolase, which is involved in the degradation of juvenile hormone (Tusun et al., 2017), was downregulated after 1 day of recovery. By the 3rd day of recovery, *CYP302A1* and *JHEH2* returned to normal levels (i.e. no significant difference in expression levels between frozen and control groups), while several pathways related to nucleic acid synthesis and repair were activated. Although the activation of some of these processes could be related to resumption of development (e.g. DNA replication and cell cycle), they could also be involved in repairing or replacing nucleic acids damaged by freezing. In addition, GO terms related to cuticular development were overrepresented among downregulated genes at day 7 and several genes coding for cuticular proteins were downregulated at days 7 and 15 after thawing. Together, by decreasing ecdysone release, keeping juvenile hormone elevated and preventing new cuticular proteins from being made, larvae could delay development to the pupal and adult stages following a freezing event. Furthermore, larvae transcriptionally inactivated metabolism specifically at day 1 of recovery. These changes were very broad, as over 30 metabolic pathways were downregulated. Considering the short growing season and considerable fine-scale microhabitat heterogeneity in Antarctica (Peck et al., 2006; Spacht et al., 2021), even short-lived developmental and metabolic shutdown may have fitness consequences for this species. For example, the adult lifespan is short (≤14 days; Usher and Edwards, 1984), and so a delayed development could cause asynchronous emergence that has negative implications for *B. antarctica*’s reproduction. Additionally, delayed adult emergence would reduce the time available for growth, which could lead to smaller body size at adulthood and potentially shorten the growing season for the next generation, thereby causing further fitness consequences for this species.

### Energy stores are not depleted throughout recovery from freezing

We hypothesized that recovery from freezing stress would cause a depletion in energy stores, as many of the repair processes discussed above are energetically costly (e.g. *de novo* transcription and translation: Lynch and Marinov, 2015; Nicholls, 2013; ATP-dependent chaperoning: King and MacRae, 2015; Kubota, 2009). Although we observed evidence of damage repair, particularly early in recovery, no significant reduction in carbohydrates, lipids or proteins was observed between frozen and control larvae at any time point. Because food was provided during the original experiment, one possible explanation for this result was that larvae immediately started feeding after thawing and replenished macronutrients during recovery, but likewise, there were no significant changes in energy stores between frozen and control larvae when food was unavailable. The dramatic upregulation of energetically costly stress-response pathways coupled with no significant decreases in energy stores is somewhat paradoxical. The most likely explanation is that the observed shutdown in metabolism during recovery minimizes energy expenditure by other processes, so there are no net changes in energy relative to untreated larvae. Energetic costs of freezing also appear to depend on the developmental stage in this species. Summer larvae experience a severe decrease in energy stores when exposed to freezing (Teets et al., 2011), while winter larvae show a modest decrease in glycogen following freezing, but otherwise no energetic consequences (Teets et al., 2020). These two studies suggest that freezing has different costs for larvae tested during the Antarctic summer and winter. Indeed, *B. antarctica* presents seasonal phenotypic variation in several traits, including tolerance to extreme freezing (Lee et al., 2006), basal metabolic rate (Spacht et al., 2020) and cryoprotectant profiles (Lee and Baust, 1981). Thus, investing in processes that resist freezing injury during the transition between summer and winter could explain why there is a disparity between energetic costs of freezing in summer and winter larvae. Future studies comparing molecular mechanisms through which winter and summer larvae recover from freezing stress could address this question and perhaps explain how the observed stress responses are accompanied by negligible energetic costs.

### Conclusions

**Fig. 4.**
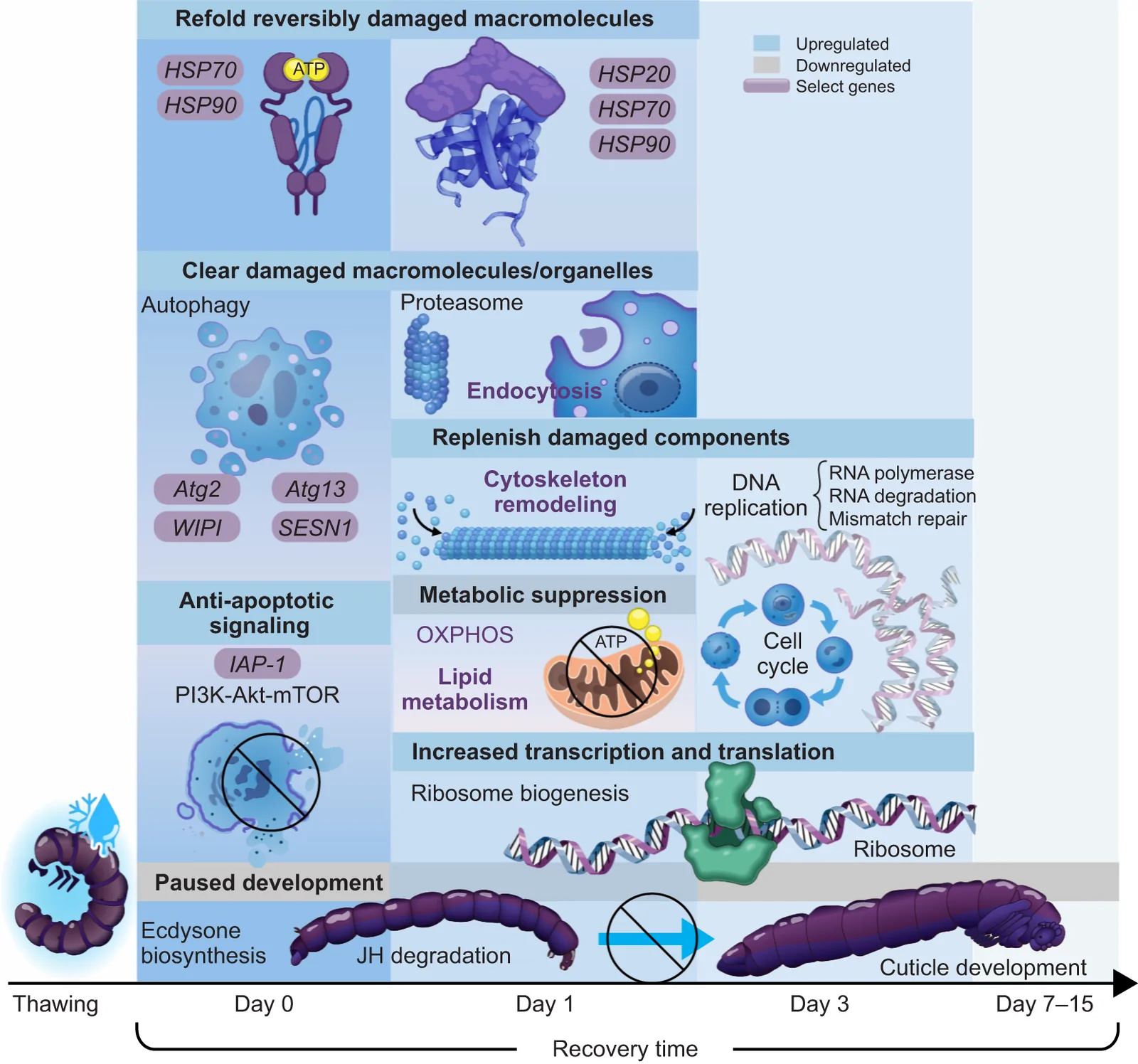
**Conceptual model of long-term recovery from freezing in *B. antarctica.*** The figure shows hypothesized major physiological responses to a freezing event occurring immediately after thawing and at days 1, 3, 7 and 15 of recovery. Blue boxes represent physiological responses that were activated and/or had increased activity, and gray boxes represent processes that had decreased activity and/or were suppressed. Purple boxes represent select genes hypothesized to be critical for recovery from freezing. Processes in bold purple (endocytosis, cytoskeletal remodeling, OXPHOS, lipid metabolism) had supporting evidence that agreed in all our approaches (i.e. differential expression, GO and KEGG). JH, juvenile hormone.

The ability to survive freezing is critical for organisms in terrestrial Antarctica, where freezing events can occur at any time of the year. *Belgica antarctica* responded to injury caused by extracellular ice formation by temporarily pausing development while increasing the expression of hundreds of genes involved in clearing cellular components and debris (e.g. autophagy, endocytosis) and mitigating and repairing protein damage (e.g. *HSP*s). These changes were accompanied by increased activity of the transcriptional and translational machinery (e.g. RNA polymerase, ribosome biosynthesis), perhaps to replenish macromolecules and cellular components (e.g. cytoskeletal remodeling). Furthermore, in conjunction with these repair processes, there was strong evidence that cell death is blocked, which likely delays or prevents unnecessary cell mortality while providing sufficient time for freezing injury to be fully addressed (Fig. 4). Future studies in other insects should investigate whether evoking this coordinated ‘emergency’ system that occurs early in recovery is a specific adaptation for *B. antarctica* or whether it is consistent across insects. In general, molecular responses agreed with our predictions, although contrary to our expectations, they did not result in a reduction of energy stores. While our data indicate that freezing poses no long-term energetic consequences to *B. antarctica*, an additional investigation is needed to determine whether there are nonetheless fitness consequences to activating these stress responses.

## Supplementary Material

10.1242/jexbio.252542_sup1 Supplementary information

Dataset 1. Functional annotation.

Dataset 2. Summaries, DEGs, GO and KEGG.
