# Supplementary material for: Molecular mechanisms and energetic costs of recovery from freezing in the polyextremophile midge Belgica antarctica
Source: J Exp Biol. 2026 Aug 12;229(15):jeb252542. doi: 10.1242/jeb.252542 (PMC13535103; doi:10.1242/jeb.252542)
Supplement: Supplementary file 1 [file jexbio-229-252542-s1.pdf]

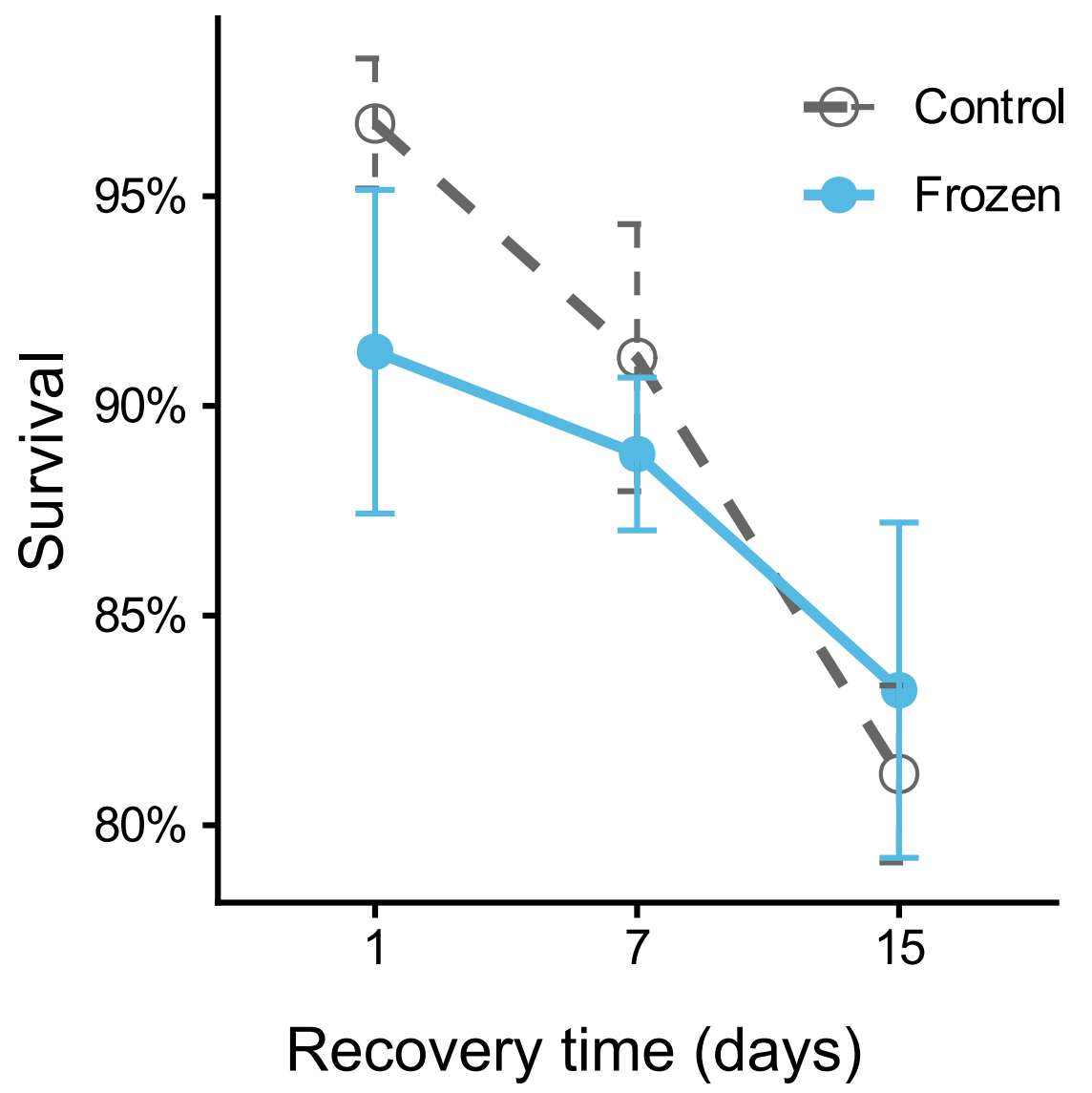

Fig. S1. Survival during recovery from extracellular freezing when food was not provided. *Belgica antarctica* larvae were either frozen at -5°C or maintained at 4°C for 24 h and survival was assessed at 1, 7, 15 days following treatment. We assessed survival of 25 larvae per replicate (n = 5 replicates), per treatment, per timepoint (total n = 600 larvae). Plot shows mean (± s.e.m.) proportion (displayed as %) of larvae that survived to treatment at each timepoint.

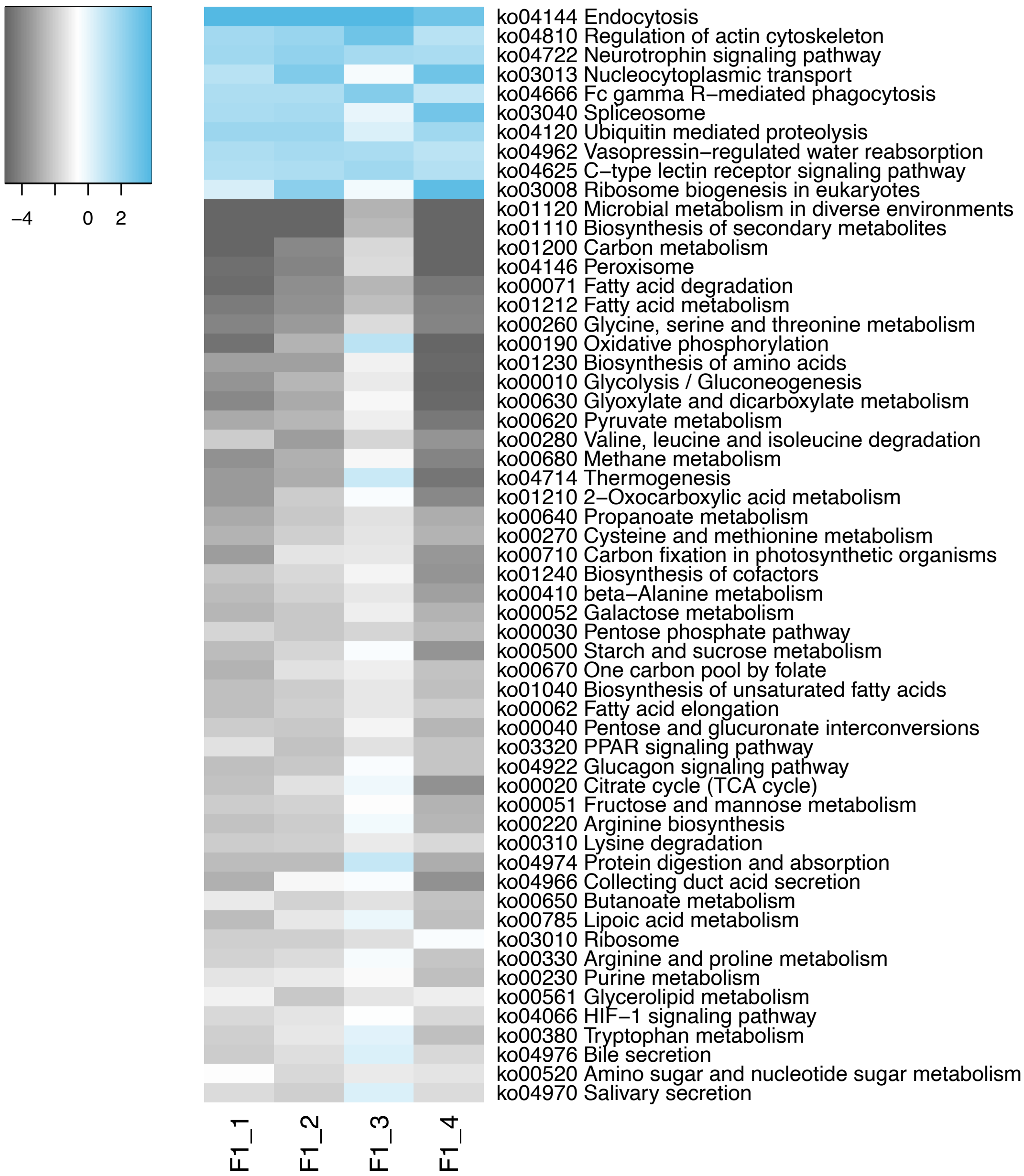

Fig. S2. Heatmap of significant KEGG pathways at timepoint 1 d identified by GAGE analysis (FDR $p < 0.05$). Each row represents a pathway. Each column represents a biological replicate (F1_1-4). Each box/cell represents the relative expression of genes between frozen and control *B. antarctica* larvae that were enriched for a given pathway. The colors indicate the pathway-level GAGE test statistic, which quantify gene expression contribution to the enrichment of the respective pathway. Positive values (blue) indicate over-representation and negative values (grey) indicate under-representation of each pathway in frozen vs. untreated larva

Journal of Experimental Biology • Supplementary information

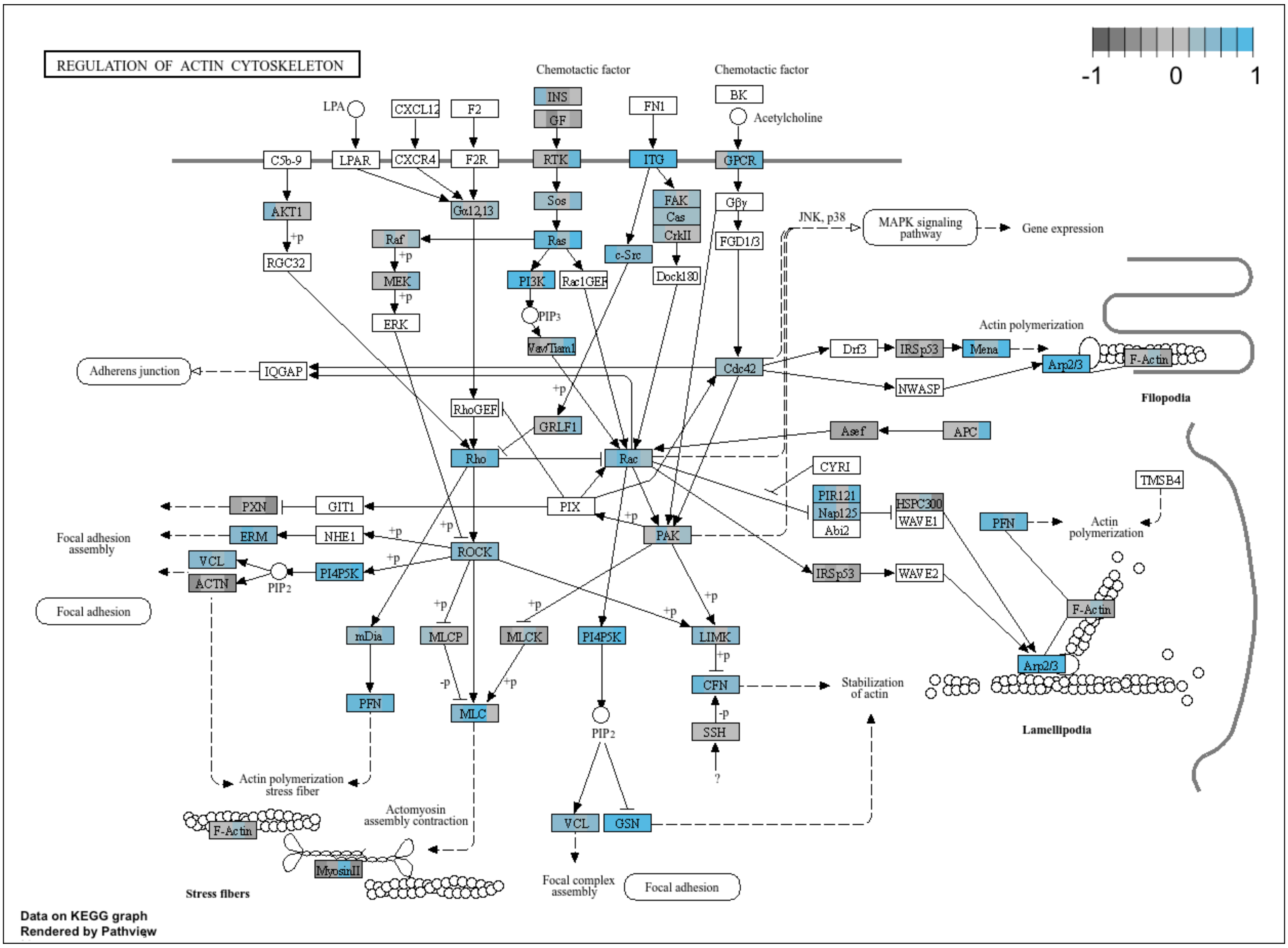

Fig. S3. Shifts in the expression of genes that enriched the regulation of actin cytoskeleton KEGG pathway (ko04810) in *Belgica antarctica* larvae at 1 d of recovery. Blue boxes represent increased expression, grey boxes represent decreased expression, and white boxes are components with unchanged expression. For a complete description of each pathway component, see https://www.kegg.jp/pathway/dme04810)

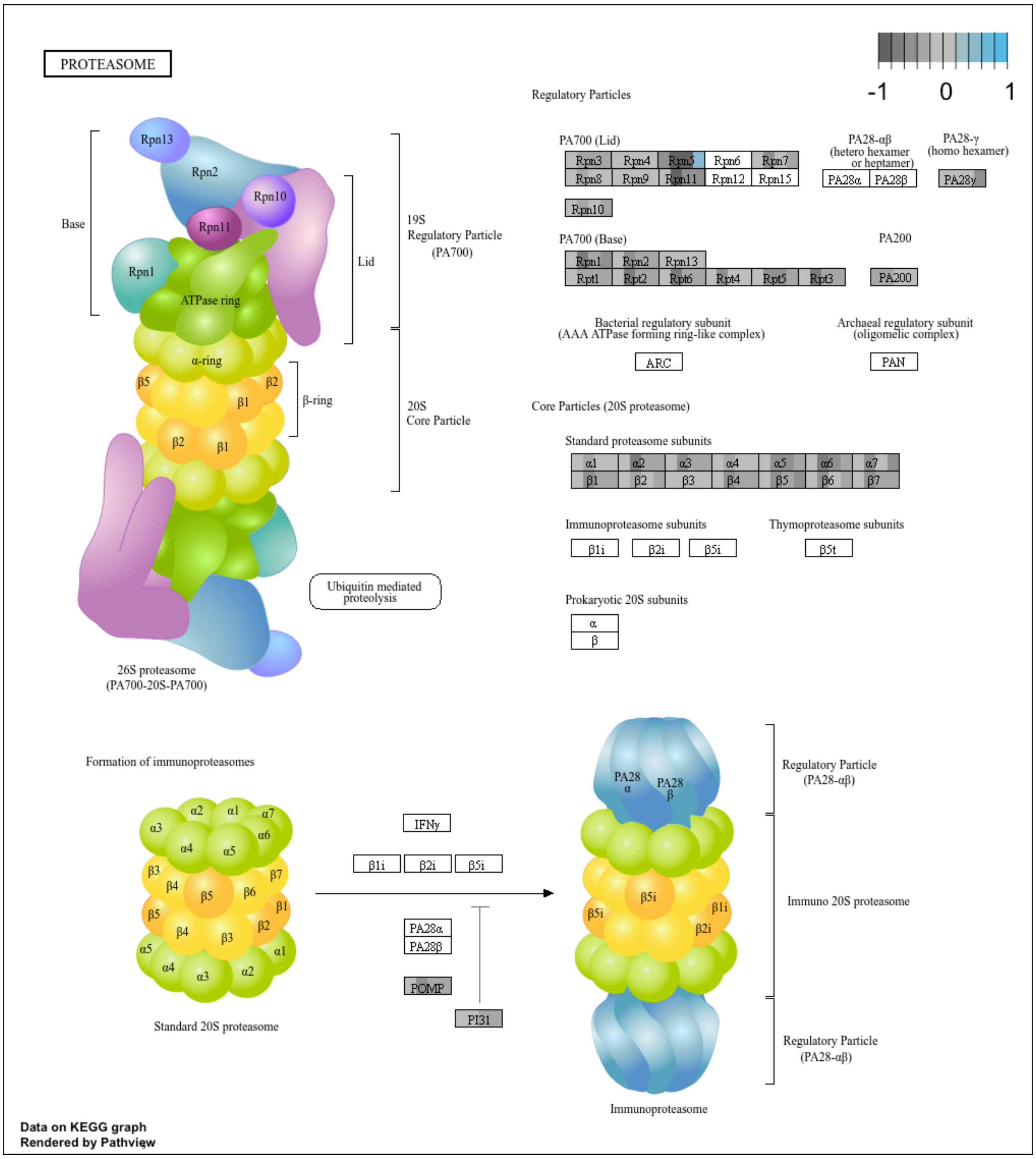

Fig. S4. Shifts in the expression of genes that enriched the regulation of the proteasome KEGG pathway (ko03050) in *Belgica antarctica* larvae at 0 d of recovery. Blue boxes represent increased expression, grey boxes represent decreased expression, and white boxes are genes that expression was unchanged. For a complete description of each pathway component, see https://www.kegg.jp/pathway/dme03050).

Journal of Experimental Biology • Supplementary information

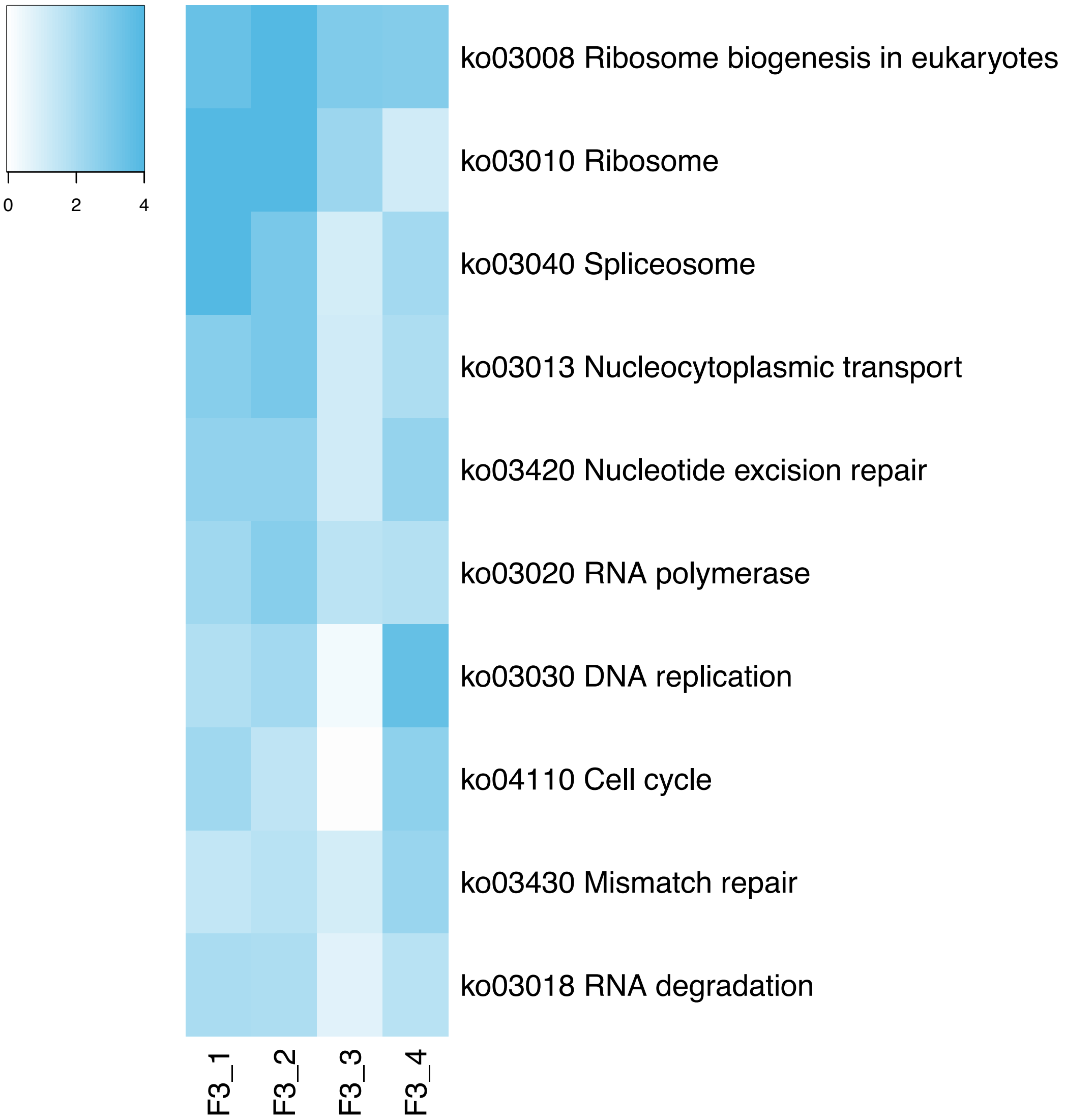

Fig. S5. Heatmap of significant KEGG pathways at day 3 identified by GAGE analysis (FDR $p < 0.05$). Each row represents a pathway. Each column represents a biological replicate (F3_1-4). Each box/cell represents the relative expression of genes between frozen and control *B. antarctica* larvae that were enriched for a given pathway (only upregulated at this timepoint). The colors indicate the pathway-level GAGE test statistic, which quantify gene expression contribution to the enrichment of the respective pathway. Positive values (blue) indicate over-representation of each pathway in frozen vs. untreated larvae.

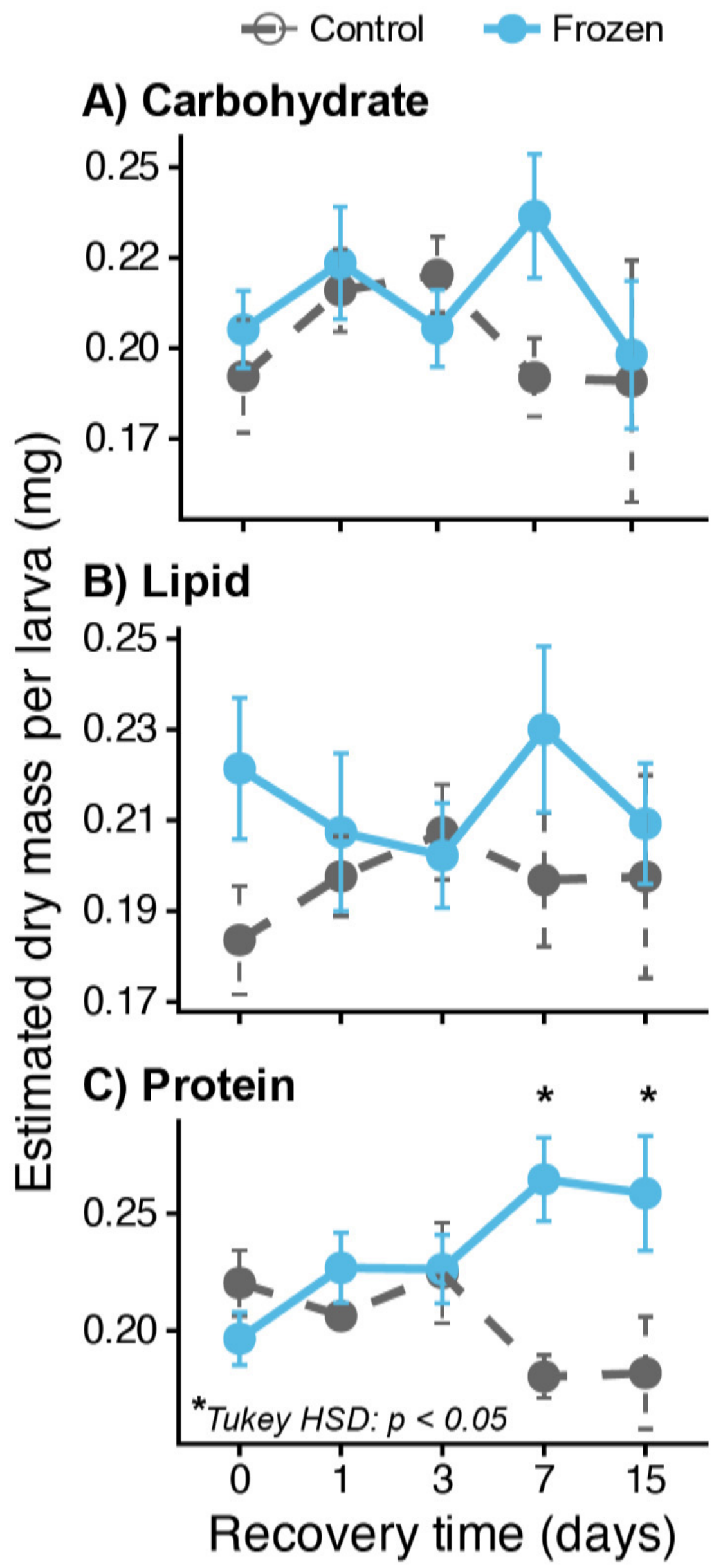

Fig. S6. Changes in estimated dry mass during recovery from extracellular freezing when food was provided during recovery. *Belgica antarctica* larvae were either frozen at -5°C or maintained at 4°C for 24 h and mass per larva was assessed at 0, 1, 3, 7 and 15 days following treatment. For each assay, we used 5 replicate pools (5 larvae per replicate), per treatment, per timepoint (total n = 50 pooled samples per macronutrient) and protocols. Plots indicate mean ± s.e.m. (mg). Plots indicate mean ± s.e.m. (mg). Factors that significantly affected response variables (Tukey HSD, p < 0.05) are indicated in each panel with asterisks.

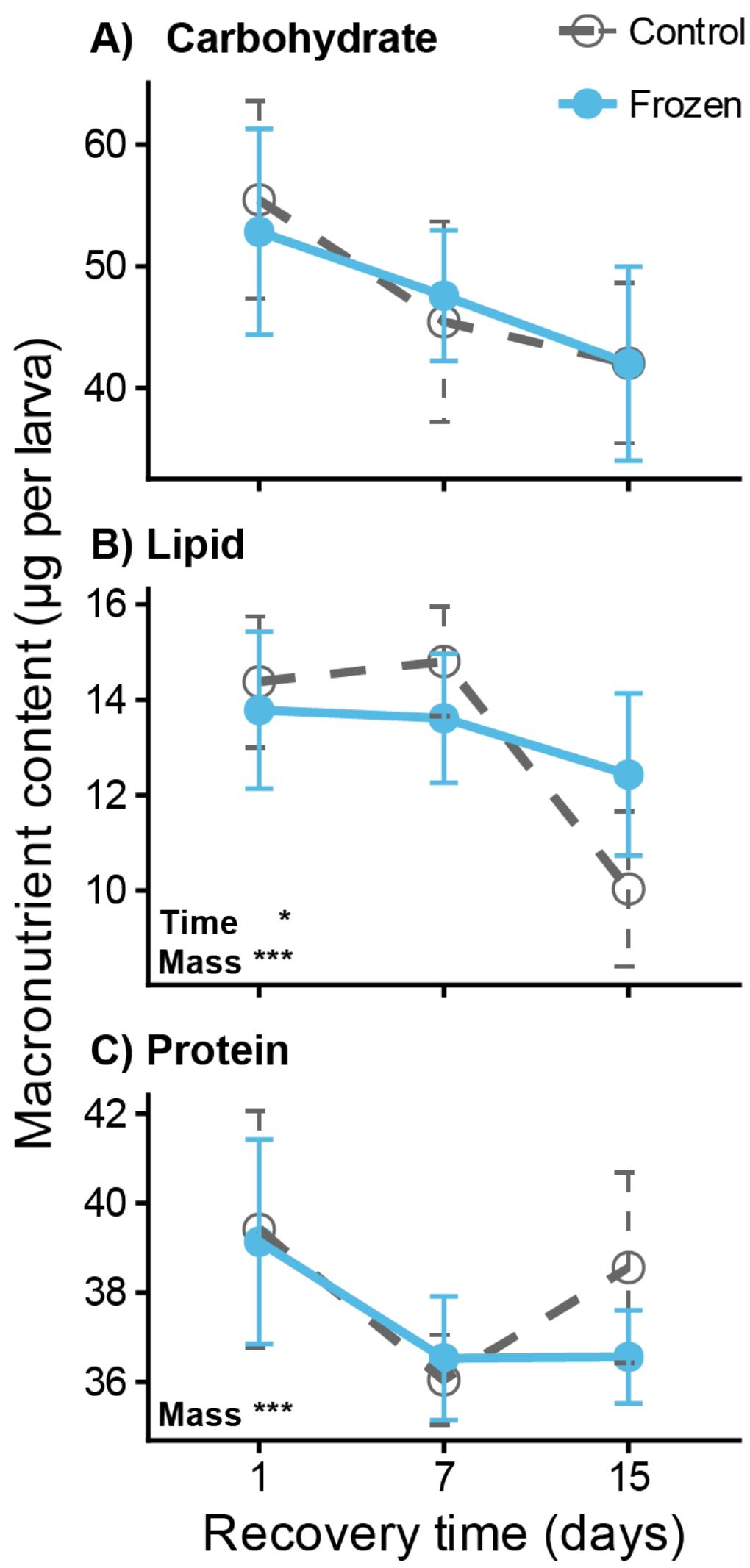

Fig. S7. Changes in energy stores during recovery from extracellular freezing when no food was provided during recovery. *Belgica antarctica* larvae were either frozen at - 5°C or maintained at 4°C for 24 h and total macronutrient content was assessed at 1, 7 and 15 days following treatment. For each assay, we used 5 replicate pools (5 larvae per replicate), per treatment, per timepoint (total n = 30 pooled samples per macronutrient) and protocols. Plots indicate mean ± s.e.m. (mg). Plots indicate mean ± s.e.m. (µg per larva). Factors that significantly affected response variables are indicated in each panel.

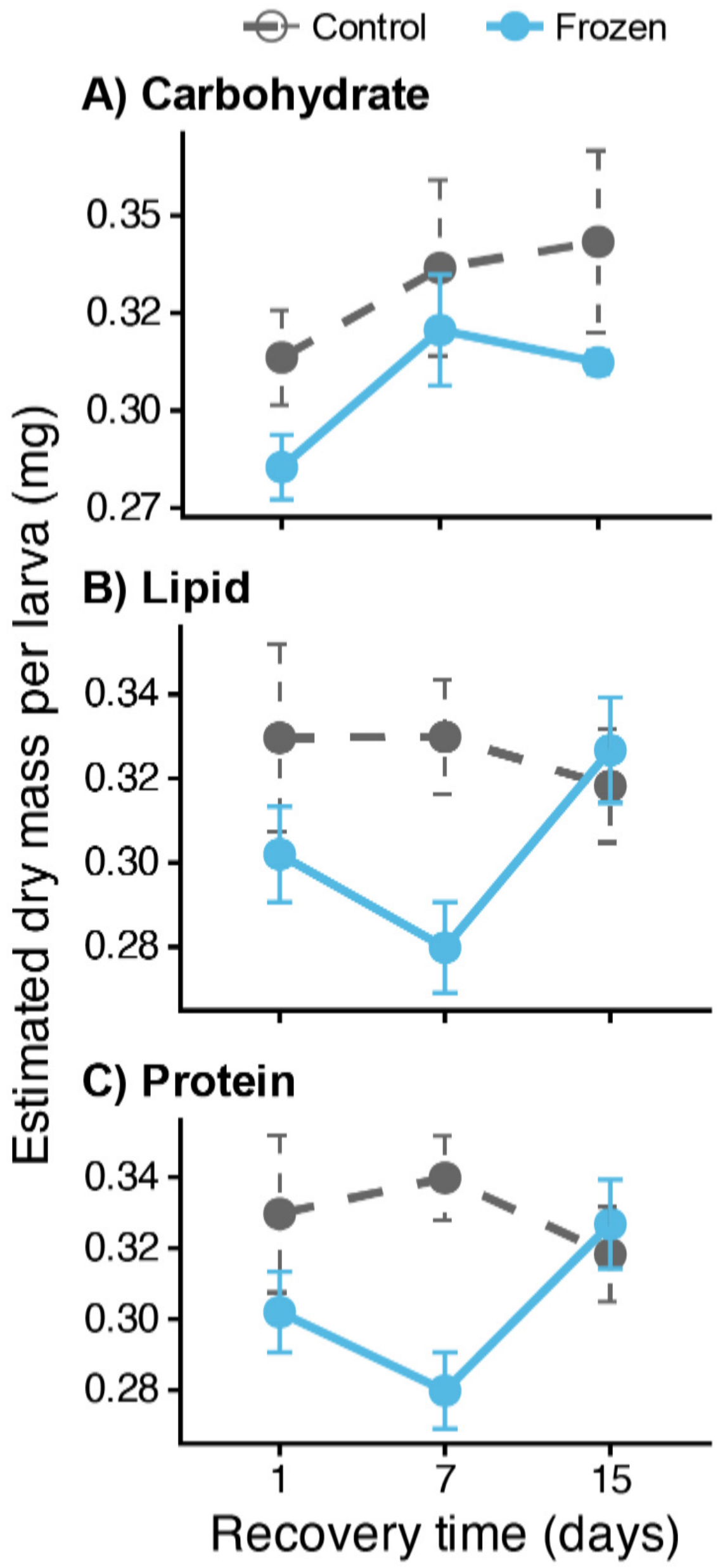

Fig. S8. Changes in estimated dry mass during recovery from extracellular freezing when no food was provided during recovery. *Belgica antarctica* larvae were either frozen at -5°C or maintained at 4°C for 24 h and mass per larva was assessed at 1, 7 and 15 days following treatment. For each assay, we used 5 replicate pools (5 larvae per replicate), per treatment, per timepoint (total n = 30 pooled samples per macronutrient) and protocols. Plots indicate mean ± s.e.m. (mg). No significant difference was detected in mass over time or between treatments (LM, ANOVA, $p > 0.05$).

Journal of Experimental Biology • Supplementary information

Dataset 1. Functional annotation. Dataset contains information on *B. antarctica* genes, including functional descriptions, GO terms and KEGG IDs when available. Dataset can be found with the online version of this manuscript.

Available for download at
https://journals.biologists.com/jeb/article-lookup/doi/10.1242/jeb.252542#supplementary-data

Dataset 2. Summaries, DEGs, GO and KEGG. Dataset contains 18 sheets. Sheet 1 presents the summary tables for number of DEGs, GO terms, KEGG pathways at each timepoint for frozen relative to control groups. Sheet 1 also contains mean (± s.e.m.) proportion of survival and macronutrient content for each pooled frozen and control group at each timepoint. Sheets 2-5 contain all DEGs, only significant DEGs (at $q < 0.05$), significant GO terms and significant KEGG pathways identified at timepoint 0 d in frozen relative to control groups. Sheets 6-9 contain all DEGs, only significant DEGs (at $q < 0.05$), significant GO terms and significant KEGG pathways identified at timepoint 1 d in frozen relative to control groups. Sheets 10-13 contain all DEGs, only significant DEGs (at $q < 0.05$), significant GO terms and significant KEGG pathways identified at timepoint 3 d in frozen relative to control groups. Sheets 14-16 contain all DEGs, only significant DEGs (at $q < 0.05$) and significant GO terms identified at timepoint 7 d in frozen relative to control groups. No differentially regulated KEGG pathways were observed between treatments at timepoint 7 d. Sheets 17-18 contain all DEGs and only significant DEGs (at $q < 0.05$) identified at timepoint 15 d in frozen relative to control groups. No overrepresented GO terms or differentially regulated KEGG pathways were observed between treatments at timepoint 15 d.

Available for download at
https://journals.biologists.com/jeb/article-lookup/doi/10.1242/jeb.252542#supplementary-data

Journal of Experimental Biology • Supplementary information

Table S1. Information on RNA-seq data from *Belgica antarctica* obtained for genome annotation.

NCBI Accession	No. of samples	Life stage	Reference
PRJNA576639	35	Adults	Finch et al., 2020
PRJNA1405672	45	Larvae	Unpublished
PRJNA1417776	36	Larvae	Unpublished
PRJNA1418125	35	Larvae	Unpublished
PRJNA1413385	40	Larvae	This study

Table S2. TaxID, species name, number of annotated protein sequences, and number of orthologues between *B. antarctica* and each closely related species.

TaxID	Species	Family	No. of protein sequences	Orthologues
7165	*Anopheles gambiae*	Culicidae	28317	2429
7159	*Aedes aegypti*	Culicidae	30505	2241
315563	*Belgica antarctica*	Chironomidae	16444	-
568069	*Clunio marinus*	Chironomidae	22577	614
315576	*Chironomus riparius*	Chironomidae	16637	770
7227	*Drosophila melanogaster*	Drosophilidae	30802	3270
319348	*Polypedilum vanderplanki*	Chironomidae	19244	973

Table S3. Summary statistics of the genome assembly and annotation and read alignments.

**Genome assembly**	
Genome size	101.19 Mbp
Number of contigs (and scaffolds)	126
N50	3,252,840 bp (3.25 Mbp)
L50	11
GC%	38.99%
**QC**	
Complete BUSCOs	1332 (99.0%)
Complete (single copy) BUSCOs	1297 (97.4%)
Complete (duplicated) BUSCOs	16 (1.2%)
Fragmented BUSCOs	4 (0.29%)
Missing BUSCOs	14 (1.02%)
**Functional annotation (eggNOG)**	
Total number of predicted protein sequences inputted	16444
Returned protein sequences with eggNOG orthologs	13999
Orthologs with a description available	13281
Protein sequences with GO Terms	9790
Protein sequences with KEGG IDs	9038
**Read alignments**	
Number of libraries	40
Number of raw 150-bp paired-end reads	1,055,598,894
Average per sample	26,389,972
Number of trimmed/cleaned reads	1,014,998,937 (96%)
Average per sample	25,374,973
Number of reads aligned to the genome (overall alignment rate)	1,013,494,978 (95.27%)
Average per sample	25,337,374

Journal of Experimental Biology • Supplementary information

Aligned concordantly 0 times (rate)	1789958 (7.04%)
Aligned concordantly exactly 1 time (rate)	22153377 (87.45%)
Aligned concordantly > 1 times (rate)	1394038 (5.50%)

Mbps: megabase pairs; bp: base pairs; N50: weighted median statistic - the minimum number of sequences needed to cover 50% of the assembly; L50, rank-order count statistic: the minimum number of sequences needed to cover 50% of the assembly; GC %; the proportion of guanine (G) and cytosine (C) bases among all bases.

Table S4. Number of DEGs, enriched KEGG pathways and overrepresented GO terms at q < 0.05.

Recovery time (days)	DEG (down)	DEG (up)	KEGG (down)	KEGG (up)	GO (down)	GO (Up)
0	61	160	1	2	0	45
1	348	485	47	10	78	629
3	9	14	0	10	0	9
7	55	10	0	0	9	0
15	10	9	0	0	0	0

Journal of Experimental Biology • Supplementary information
